# Perceptual History Biases Are Predicted by Early Visual-Evoked Activity

**DOI:** 10.1523/JNEUROSCI.1451-22.2023

**Published:** 2023-05-24

**Authors:** Michele Fornaciai, Irene Togoli, Domenica Bueti

**Affiliations:** Cognitive Neuroscience Department, International School for Advanced Studies, 34136 Trieste, Italy

**Keywords:** EEG, magnitude perception, perceptual history, serial dependence

## Abstract

What we see in the present is affected by what we saw in the recent past. Serial dependence, a bias making a current stimulus appear more similar to a previous one, has been indeed shown to be ubiquitous in vision. At the neural level, serial dependence is accompanied by a signature of stimulus history (i.e., past stimulus information) emerging from early visual-evoked activity. However, whether this neural signature effectively reflects the behavioral bias is unclear. Here we address this question by assessing the neural (electrophysiological) and behavioral signature of stimulus history in human subjects (both male and female), in the context of numerosity, duration, and size perception. First, our results show that while the behavioral effect is task-dependent, its neural signature also reflects task-irrelevant dimensions of a past stimulus, suggesting a partial dissociation between the mechanisms mediating the encoding of stimulus history and the behavioral bias itself. Second, we show that performing a task is not a necessary condition to observe the neural signature of stimulus history, but that in the presence of an active task such a signature is significantly amplified. Finally, and more importantly, we show that the pattern of brain activity in a relatively early latency window (starting at ∼35-65 ms after stimulus onset) significantly predicts the behavioral effect. Overall, our results thus demonstrate that the encoding of past stimulus information in neural signals does indeed reflect serial dependence, and that serial dependence occurs at a relatively early level of visual processing.

**SIGNIFICANCE STATEMENT** What we perceive is determined not only by the information reaching our sensory organs, but also by the context in which the information is embedded. What we saw in the recent past (perceptual history) can indeed modulate the perception of a current stimulus in an attractive way, a bias that is ubiquitous in vision. Here we show that this bias can be predicted by the pattern of brain activity reflecting the encoding of past stimulus information, very early after the onset of a stimulus. This in turn suggests that the integration of past and present sensory information mediating the attractive bias occurs early in the visual processing stream, and likely involves early visual cortices.

## Introduction

Visual perception is not uniquely based on the sensory input received at any given moment but also reflects the influence of the recent history of stimulation, or perceptual history. An increasing amount of evidence indeed shows that the perception and judgment of a current stimulus are modulated by the stimuli seen in the recent past. Namely, a current stimulus is perceived to be more similar to its preceding one than it actually is—an “attractive” bias that has been named serial dependence (e.g., Fischer and Whitney, 2014). This bias is thought to originate from the integration of past and present sensory evidence (e.g., Burr and Cicchini, 2014; Fischer and Whitney, 2014), and has been shown to be ubiquitous in vision (Cicchini et al., 2014; Liberman et al., 2014; Alais et al., 2017; Fritsche et al., 2017; Manassi et al., 2018).

However, the nature of serial dependence and its neural mechanisms are still unclear. This attractive effect has been indeed proposed to engage different putative mechanisms, spanning from sensory or perceptual processing (e.g., Fischer and Whitney, 2014; Fornaciai and Park, 2018a, 2020a) to a completely postperceptual mechanism based on memory or decision (e.g., Fritsche et al., 2017; Wehrman et al., 2020). Overall, although serial dependence seems to operate at the perceptual level (Cicchini et al., 2017; Fritsche and de Lange, 2019), it also shows the hallmarks of high-level visual processes (e.g., Fornaciai and Park, 2018a, 2021, 2022; Pascucci et al., 2019; Samaha et al., 2019), making it indeed difficult to understand its nature.

At the neural level, previous studies mostly provide evidence supporting the idea that serial dependence is perceptual in nature. For instance, St John-Saaltink et al. (2016), using fMRI, showed that the recent history of stimulation biases orientation representations directly in the primary visual cortex. Additionally, EEG results further support the idea of an early signature of serial dependence (Fornaciai and Park, 2018a, 2019b), showing that stimulus history is decodable from visual-evoked potentials early on after the onset of a stimulus, compatibly with the timing of activity in early visual areas (e.g., Di Russo et al., 2005; Fornaciai et al., 2017). However, whether such early signature of stimulus history actually represents a genuine correlate of the behavioral serial dependence effect is unclear.

In the present study, we address the link between the behavioral attractive effect entailed by serial dependence and the neural signature of stimulus history, aiming to pinpoint the brain processing stages involved with the behavioral bias. In Experiment 1, we used EEG in conjunction with different magnitude discrimination tasks (i.e., numerosity, duration, and size discrimination task, in three different conditions). Namely, participants had to discriminate the numerosity, the duration, or the dot size of a constant reference dot-array stimulus compared with a variable probe array. To induce serial dependence effects, we presented a task-irrelevant “inducer” stimulus (always modulated in numerosity, duration, and dot size in all task conditions) before the task-relevant ones, and assessed (1) how the inducer affects the perceived magnitude of the reference and (2) how the inducer magnitude information is carried over to the reference processing at the neural level. Following previous studies, we computed the extent to which the past (inducer) stimulus information is encoded in brain signals using a multivariate “decoding” analysis (e.g., King and Dehaene, 2014). In Experiment 2, we instead used a passive viewing paradigm, to address the potential role of task relevance of the stimuli in driving the neural signature of stimulus history. In this experiment, the participants simply watched a sequence of dot-array stimuli modulated in numerosity, duration, and size while responding to occasional odd-ball stimuli defined by contrast (i.e., to avoid making the magnitude dimensions task relevant as in Experiment 1 while ensuring that the subjects attended the stimuli).

## Materials and Methods

### Subjects

A total of 63 subjects took part in the study (42 females; mean age = 23.9 years, SD = 3.8 years; including the author I.T.): 32 of them were tested in Experiment 1, and 31 were tested in Experiment 2. Two subjects in Experiment 1 were excluded because of noisy EEG recordings (see Electrophysiological recording and analysis). Two subjects were instead excluded from Experiment 2 because of equipment failure (missing EEG data). The final samples included in data analysis were thus 30 and 29 subjects in Experiments 1 and 2, respectively. All participants had normal or corrected-to-normal vision, were naive to the purpose of the study (with the exception of one of the authors who participated in Experiment 1), and signed a written informed consent form before taking part in the study. All experimental procedures were approved by the ethics committee of the International School for Advanced Studies, and were in line with the Declaration of Helsinki.

The sample size of the experiments was based on a previous behavioral study from our group examining the serial dependence effect in duration and numerosity perception (Togoli et al., 2021). More specifically, we estimated an average effect size (Cohen's *d*) from the numerosity and duration discrimination tasks tested in Togoli et al. (2021) equal to 0.55. Assuming a one-tailed distribution (based on our hypothesis concerning the direction of the effect) and a desired power of 0.9, a power analysis indicated a minimum sample size of 30 participants. In addition to the power analysis based on the behavioral effects, we also considered the effect size obtained in previous EEG studies (Fornaciai and Park, 2019b). The effect size (Cohen's *d*) in this case was estimated to be 0.6. We thus decided to base the sample sizes on the more conservative estimate based on previous behavioral results.

### Apparatus and stimuli

The experiments were performed in a quiet and dimly lit boot, equipped with a Faraday cage. Stimuli were presented on a 1920 × 1080 monitor screen running at 120 Hz, positioned ∼80 cm from the participant. All the stimuli were generated using the Psychophysics Toolbox (Pelli, 1997; Kleiner et al., 2007) on MATLAB (version r2019b; The MathWorks). In all the experimental conditions, stimuli were arrays of black and white dots (50%/50% proportion) presented on a gray background, randomly positioned within a circular area (i.e., field area). In Experiment 1, a sequence of three dot-array stimuli was presented on the screen in each trial. The first stimulus in the sequence was a task-irrelevant “inducer” stimulus used to induce serial dependence. The inducer stimulus could contain either 12 or 24 dots (numerosity), could be presented for either 140 or 280 ms (duration), and contained dots with radius equal to 4 or 8 pixels (dot size). The levels of the different magnitude dimensions of the inducer were identical in all the task conditions of Experiment 1. The second stimulus was a reference stimulus that was kept constant across all the trials and task conditions. The reference always contained 16 dots, was presented for 200 ms, and each dot had a radius equal to 6 pixels. The last stimulus in the sequence was a probe stimulus that varied according to the specific task condition. Namely, in the numerosity task, the probe contained 8, 12, 16, 24, or 32 dots, had a duration of 200 ms, and dot size of 6 pixels. In the duration task, the probe had constant numerosity (16 dots) and dot size (6 pixels), and varied in duration (100, 140, 200, 280, or 400 ms). In the size task, the probe had constant numerosity (16 dots) and duration (200 ms), and was varied in dot size (3, 4, 6, 8, or 12 pixels). In Experiment 2, a single stimulus was presented in each trial. All the stimuli were thus modulated in numerosity, duration, and dot size, according to three levels for each dimension. Namely, each dot array could contain 12, 16, or 24 dots, could be presented for 140, 200, or 280 ms, and had dot size of 4, 6, or 8 pixels. Occasionally (10 trials in each block of 270 trials; 3.7% of the trials), a “catch” stimulus was presented, which was characterized by a reduced contrast (30% less compared with standard stimuli). In each experiment and condition, the field area of the dot-array stimuli was randomly modulated spanning from 200 to 400 pixels in radius.

### Experimental design

In Experiment 1, participants performed three different discrimination tasks in separate conditions and in a random order (i.e., numerosity, duration, or size discrimination task, involving a 2-alternative forced-choice procedure). The stimulation sequence was largely identical across conditions. Namely, a sequence of three stimuli was presented in each trial: an inducer stimulus, followed by a reference (inducer-reference interstimulus interval [ISI] = 650-850 ms), and finally a probe (reference-probe ISI = 600-650 ms). While the inducer and reference stimuli were identical in all the tasks (see Apparatus and stimuli), the probe was modulated according to the task; that is, it varied in numerosity in the numerosity task, in duration in the duration task, and in dot size in the size task. At the end of the stimulus sequence, participants had to report which stimulus between the reference and the probe contained more dots, lasted longer in time, or contained larger dots. Participants were instructed to respond as fast and accurately as possible; and although the inducer was irrelevant for the task, to pay anyway attention to the entire sequence of the stimuli, in line with previous studies (e.g., Fornaciai and Park, 2018b). The available time to provide a response was limited to 1250 ms. Once a participant provided a response, the next trial started automatically after 800-1200 ms. If no response was provided within 1250 ms from the offset of the last stimulus in the sequence, the next trial started automatically. Responses were collected by means of a standard keyboard. On average, participants missed the response in 11 (SD = 15.7) trials of a total of 400 trials performed in each condition. Trials in which no response was provided were excluded from behavioral data analysis but included in the EEG analysis.

In Experiment 2, instead, participants were asked to watch a continuous stream of dot-array stimuli modulated in numerosity, duration, and dot size. The ISI between consecutive stimuli was 800 ms. To encourage participants to pay attention to the stimulus sequence, we asked them to perform a simple oddball detection task. More specifically, an occasional oddball (“catch”) stimulus was presented (3.7% of the trials), with a reduced contrast compared with the majority of other (“standard”) stimuli. Participants were instructed to press a button on a keyboard as fast as they could once they detected the lower-contrast oddball stimulus. The detection rates in this task were on average (±SD) 93 ± 1.3%. The average reaction time in correctly detected catch trials was 313 ± 11 ms.

In Experiment 1, participants completed 10 blocks of 40 trials in each task condition, including 20 repetitions of each combination of inducer and probe magnitude. In Experiment 2, participants completed 8 blocks of 270 trials, for a total of 80 repetitions of each combination of stimulus magnitudes. Participants were free to take breaks between different blocks. The Experiment took ∼1 h in the case of Experiment 1, and ∼50 min in the case of Experiment 2.

### Behavioral data analysis

In Experiment 1, participants' performance in the three different tasks was analyzed to assess to what extent the perceived magnitude of the reference stimulus was affected by the inducer. First, the proportion of responses in the task obtained from each participant and condition was fitted with a cumulative Gaussian function according to the maximum likelihood method (Watson, 1979). The point of subjective equality (PSE), representing the perceived magnitude of the reference stimulus (i.e., accuracy), was defined as the median of the cumulative Gaussian function. As a measure of precision in the task, we computed the just noticeable difference (JND) as the difference in probe magnitude between chance level response (50%) and 75% “probe more numerous/longer/bigger” responses. As an additional measure of precision, we computed the Weber fraction as JND/PSE. Finally, during the fitting procedure, we applied a finger error rate correction (5%) to account for random errors and lapses of attention (Wichmann and Hill, 2001). The PSE and the JND were computed as a function of the different levels of the inducer magnitudes, separately for each dimension (i.e., the numerosity, duration, and size of the inducer). As a final estimate of the precision, we considered the average WF across the different levels of the inducer magnitudes in each task. The difference in PSE obtained with different inducer magnitudes within each task (shown in Fig. 2*A*) was assessed with a series of paired *t* tests. To control for multiple comparisons, we used a false discovery rate (FDR) procedure, with *q* = 0.05. When reporting series of *t* tests, we thus report the *p* value adjusted by the FDR procedure (“adj-*p*”). The difference in WF across the tasks was assessed with a one-way repeated-measures ANOVA, with factor “task.”

Moreover, to better compare the serial dependence effects obtained in different tasks, we computed a serial dependence effect index according to the following formula:

$$\text{Serial dependence effect }=\left(\left({\text{PSE}}_{\text{high}}–{\text{ PSE}}_{\text{low}}\right)/{\text{PSE}}_{\text{low}}\right)\text{ × 1}00$$

Where PSE_low_ refers to the PSE obtained when the inducer magnitude was low (i.e., 12 dots in the numerosity task, 140 ms in the duration task, 4 pixels in the size task), while PSE_high_ refers to PSEs obtained with a high inducer magnitude (24 dots, 280 ms, or 8 pixels, according to the task). This index was calculated separately for each participant and condition, and the average is shown in the Figure 2*B*. Additional analyses (data not shown) were performed to assess whether the influence of the inducer is limited to the immediately following reference stimulus, or whether it extends across trials. To do so, we computed the PSE and the serial dependence effect as a function of the magnitudes of the inducer in the preceding trial or two trials back in the past. These analyses did not show any influence of the inducer across trials, most likely because of the presence of several intervening stimuli. Previous results concerning serial dependence in magnitude perception (although limited to numerosity perception) indeed showed that the effect is mostly limited to the immediately preceding stimulus (Cicchini et al., 2014; Fornaciai and Park, 2020b, 2022). As a sanity check, we also assessed whether the reference stimulus could be affected by the inducer in the successive (future) trial. No effect was observed also in this case.

To obtain an additional measure of the influence of each inducer magnitude on behavioral performance, we further performed a nonlinear regression analysis. In this analysis, performed separately for each participant and condition, we used the individual responses across trials (i.e., coded as 0 or 1, as obtained in the a 2-alternative forced-choice task procedure) as dependent variable, and included the probe magnitude (defined according to the task), inducer numerosity, inducer duration, and inducer dot size as predictors. This allowed us to directly assess the influence of each inducer magnitude level on the response in each trial. To perform this regression analysis, the levels of inducer magnitudes were coded as the ratio with the corresponding reference magnitude. Regarding the effects computed with this analysis, positive β values indicate an attractive effect (i.e., increased probability of judging the reference as “bigger” than the probe with higher inducer magnitude), while negative β values indicate a repulsive effect. The resulting β values obtained for each participant and magnitude (see Fig. 2*C*; β values corresponding to the effect of the probe are not shown in the figure) were then tested with a one-sample *t* test to assess whether the corresponding predictor provided an effect significantly >0. All statistical tests were performed in MATLAB (version R2018b).

### Electrophysiological recoding and analysis

In both Experiments 1 and 2, the EEG was recorded throughout the experimental procedure, using the Biosemi ActiveTwo system (at a sampling rate of 2048 Hz), and a 32-channel cap based on the 10–20 system layout. In Experiment 2, an electro-oculogram channel was also added below the left eye. Electrode offsets across channels were usually kept <15 µV, but occasionally offsets up to 30 µV were tolerated.

EEG data analysis was performed offline in MATLAB (version R2018b), using the EEGLAB software package (Delorme and Makeig, 2004). EEG signals were first high-pass filtered (0.1 Hz) and rereferenced to the average of all the channels used. Continuous EEG data were then segmented into epochs spanning from −200 to 700 ms after stimulus onset, with epochs time-locked to the reference stimulus in Experiment 1, or time-locked to each stimulus in Experiment 2. In order to test the influence of the inducer stimulus (Experiment 1) or the stimulus in the preceding trial (Experiment 2), epochs were also sorted as a function of the magnitudes of the preceding stimulus. For instance, in Experiment 1, the reference epochs were sorted according to the different levels of the inducer magnitudes (i.e., separately for cases were the inducer had 12 or 24 dots, was presented for 140 or 280 ms, or had dot-size of either 4 or 8 pixels). In Experiment 2, we considered as “reference” of one magnitude dimension all the stimuli presenting the intermediate magnitude level (i.e., 16 dots, 200 ms, 6 pixels, which corresponded to the reference stimulus in Experiment 1). We thus sorted epochs according to whether such intermediate stimuli (i.e., the “current” magnitude) were preceded by a stimulus having either a lower or higher magnitude, separately for each dimension (i.e., the “past” magnitude). Data from both experiments were cleaned by means of an independent component analysis, aimed to remove artifacts related to eye movements, blinks, or other sources of noise. After the independent component analysis, we used a step-like artifact rejection procedure to remove any remaining large artifact, leading to an average rejection rate of 9.8% (SD = 13.5%) in Experiment 1 and 0.6% (SD = 1.2%) in Experiment 2. Rejection rate was used as a criterion for inclusion in data analysis, with a cutoff rejection rate of 35%. Two participants in Experiment 1 were excluded from data analysis based on this criterion. Because of equipment failure, one participant in Experiment 1 had one missing block of trials but was nevertheless included in data analysis as the number of available trials was sufficient to perform the multivariate analysis. Finally, we applied a low-pass filter with a cutoff of 30 Hz.

### Multivariate pattern analysis in the time domain

In order to characterize the neural signature of stimulus history during the processing of the current stimulus, we used a multivariate pattern analysis in the time domain (or “decoding” analysis), using the Neural Decoding Toolbox (Meyers, 2013). This analysis has indeed proven to be very sensitive in decoding stimulus history in previous studies (Fornaciai and Park, 2018a, 2019b). In general, the multivariate analysis involves the training of a pattern classifier (support vector machine) on EEG data coming from multiple channels, corresponding to two specific classes of stimuli. Then, the classifier is tested on an independent subset of unlabeled data to assess whether it could discriminate the class of stimuli to which the test data belong. The classification accuracy (CA) yielded by the classifier in correctly discriminating the two classes of stimuli indicates the extent to which they generate unique patterns of activity; and by repeating this procedure across several time windows, it hence provides an index of when the stimulus information is encoded in brain activity.

To assess the neural signature of stimulus history, in Experiment 1 we used epoched EEG data time-locked to reference stimulus onset, with epochs sorted as a function of different inducer magnitudes. To assess the effect of inducer numerosity on the reference, we entered in the analysis epochs corresponding to the reference stimulus preceded by either a 12 or 24 dot inducer (regardless of the other magnitudes). Similarly, in the case of duration and size, we used epochs corresponding to the reference preceded by a 140 or 280 ms inducer, or 4 or 8 pixels inducer. The classifier was thus tested in discriminating activity evoked by an identical reference stimulus as a function of the magnitude of the preceding inducer. This analysis was performed separately for each participant, task, and inducer magnitude dimension. In Experiment 2, we used epochs corresponding to the stimuli with intermediate magnitude levels (i.e., 16 dots, 200 ms, 6 pixels) preceded by either a lower or higher magnitude across the three dimensions. For instance, we used epochs corresponding to a 16 dot stimulus preceded by either 12 or 24 dots, 140 or 280 ms, or 4 or 8 pixels, to assess the effect of different magnitudes on numerosity. The same was done for the duration and size of the stimuli.

In both experiments, we implemented a series of practices to optimize the analysis and reduce noise. First, instead of using subsets of single trials to train and test the classifier, we averaged together random sets of trials (Grootswagers et al., 2017) to create averaged “pseudo-trials.” The trials used to generate pseudo-trials were randomly drawn from the dataset, and hence were not groups of subsequent trials. The number of individual trials averaged into pseudo-trials varied between 10 and 20 based on the number of available trials after artifact rejection (on a subject-by-subject basis), with an average (±SD) of 18.1 ± 1.8 trials in Experiment 1, and 19.1 ± 2.9 in Experiment 2. The training of the classifier was thus performed on a set of such pseudo-trials, and testing was performed on a remaining pseudo-trial according to a leave-one-out procedure. The specific number of pseudo-trials used in the cross-validation procedure was fixed to 10 in Experiment 1 (i.e., nine trials for training and the remaining one for testing) and varied in Experiment 2 according to the available data (mean ± SD = 11.5 ± 2.1 pseudo-trials). To avoid overfitting (i.e., the classifier learning an overly specific pattern that fails to generalize to the test set), we also performed a feature selection procedure before the decoding analysis, restricting the decoding procedure to the five most informative EEG channels as determined with a univariate ANOVA performed on the training set (Grootswagers et al., 2017). Such feature selection procedure does not make the analysis circular. Indeed, the feature selection was performed only on the training dataset, leaving the test set independent. Since the selection of specific channels during the analysis does not provide information about how they contribute to the decoding performance, we chose not to explicitly assess the frequency of channel selection and the topography of the most frequently selected channels. Moreover, instead of performing the analysis at individual time points, we averaged activity across a series of 100 ms time windows, with a step size of 20 ms. Finally, the decoding procedure was repeated 30 times using different subsets of trials for training and testing and for creating pseudo-trials, and the average of all the iterations of the analysis was taken as the final estimate of classification performance. CA measures obtained throughout the epoch reflect to what extent the pattern classifier was able to classify stimuli according to the pattern of activity, and hence, considering our comparisons, to what extent information from the previous stimulus is encoded in the brain responses to a current one.

Decoding results were tested for significance by taking the average across two latency windows: an early window spanning from 50 to 200 ms after stimulus (based on previous studies; Fornaciai and Park, 2018a, 2019b), and a late latency window spanning from 500 to 650 ms after stimulus. This second window was chosen to span late latencies capturing postperceptual processes, such as working memory encoding and maintenance (see, e.g., Oh et al., 2019). Decoding results averaged in these two latency windows were tested using a series of one-sample *t* tests (against an empirical measure of chance level, see below), followed by a three-way repeated-measures ANOVA in both Experiments 1 and 2. In Experiment 1, we entered as factors “task” (numerosity, duration, and size task), “inducer magnitude” (numerosity, duration, and size), and “latency window” (early vs late). In Experiment 2, we entered as factors “current magnitude” (i.e., representing stimuli with the intermediate level of either numerosity, duration, or size), “past magnitude” (i.e., representing the stimuli in the immediately preceding trial with the extreme levels of either numerosity, duration, or size), and “latency window.” To assess interaction effects in these two ANOVAs, we further used simpler ANOVA models and paired *t* tests. Finally, to directly compare the decoding results obtained in the two experiments, we used a mixed-model ANOVA with “current magnitude” (i.e., either the task-relevant magnitude in Experiment 1, or the magnitude considered in the current trial in Experiment 2), “past magnitude” (i.e., the different inducer magnitudes in Experiment 1, or the corresponding different magnitudes of the preceding stimulus in Experiment 2), and “latency window” as within-subject factors, and “experiment” (Experiment 1 vs Experiment 2) as between-subject factor.

In addition to our main decoding analysis, we also performed a control (“null”) decoding analysis. In this analysis, we replicated our main procedure with the exception that we shuffled the labels of the trials in the training set before the classification procedure. The results of this null decoding analysis were then used to set the chance level empirically. One-sample *t* tests against chance level were thus performed against the corresponding average CA of the null analysis, rather than against the 50% probability level.

Finally, to address the link between the behavioral serial dependence effect and the CA obtained in the decoding procedure, we performed a series of tests based on a linear mixed-effect regression model, defined as follows:

Eff ∼ CA +(1 | Subj)

Where Eff represents the index of the behavioral serial dependence effect computed according to Equation 1, CA represents the classification accuracy of the decoding procedure, and (1 | Subj) represents the random effect (subjects). First, we performed regression tests considering the average CA in each of the two latency windows used in the analysis (50-200 ms and 500-650 ms after stimulus onset). For instance, we assessed the relation between the CA of the inducer numerosity and the behavioral effect caused by the inducer numerosity in each task, and so on for all the other inducer dimensions. Additionally, we also applied this regression model in a more comprehensive fashion, to more precisely assess the latencies at which a relationship between neural and behavioral effect might emerge. Namely, the same regression model was applied at each time window throughout the reference epoch (i.e., corresponding to the 100 ms time windows used in the decoding analysis). In this procedure, we only considered significant clusters of at least two consecutive time windows showing a significant relation between the behavioral effect and CA. To ensure the robustness of these clusters of significant time windows, we used a cluster-based nonparametric test. In this test, we examined each of the clusters identified in the main procedure separately. Namely, we shuffled the distribution of CA at each time window within the cluster and the distribution of behavioral effect across the group, and applied the same linear mixed-effect (LME) model on these shuffled data, again separately for each time window within each cluster. The procedure was repeated 10,000 times, shuffling the datasets each time and collecting the results. To assess the robustness of the actual clusters, we measured how many times the simulated cluster showed the same number of contiguous significant time windows as the actual cluster, which represents the *p* value of the test. As a threshold for determining the significance of each test performed within a simulated cluster, we used the minimum *t* value obtained in each actual cluster. All the analyses were performed in MATLAB (version r2018b).

### Data availability

The data generated during the experiments described in this manuscript is available on Open Science Framework following this link: https://osf.io/ju78r/.

## Results

### Experiment 1

**Figure 1.**
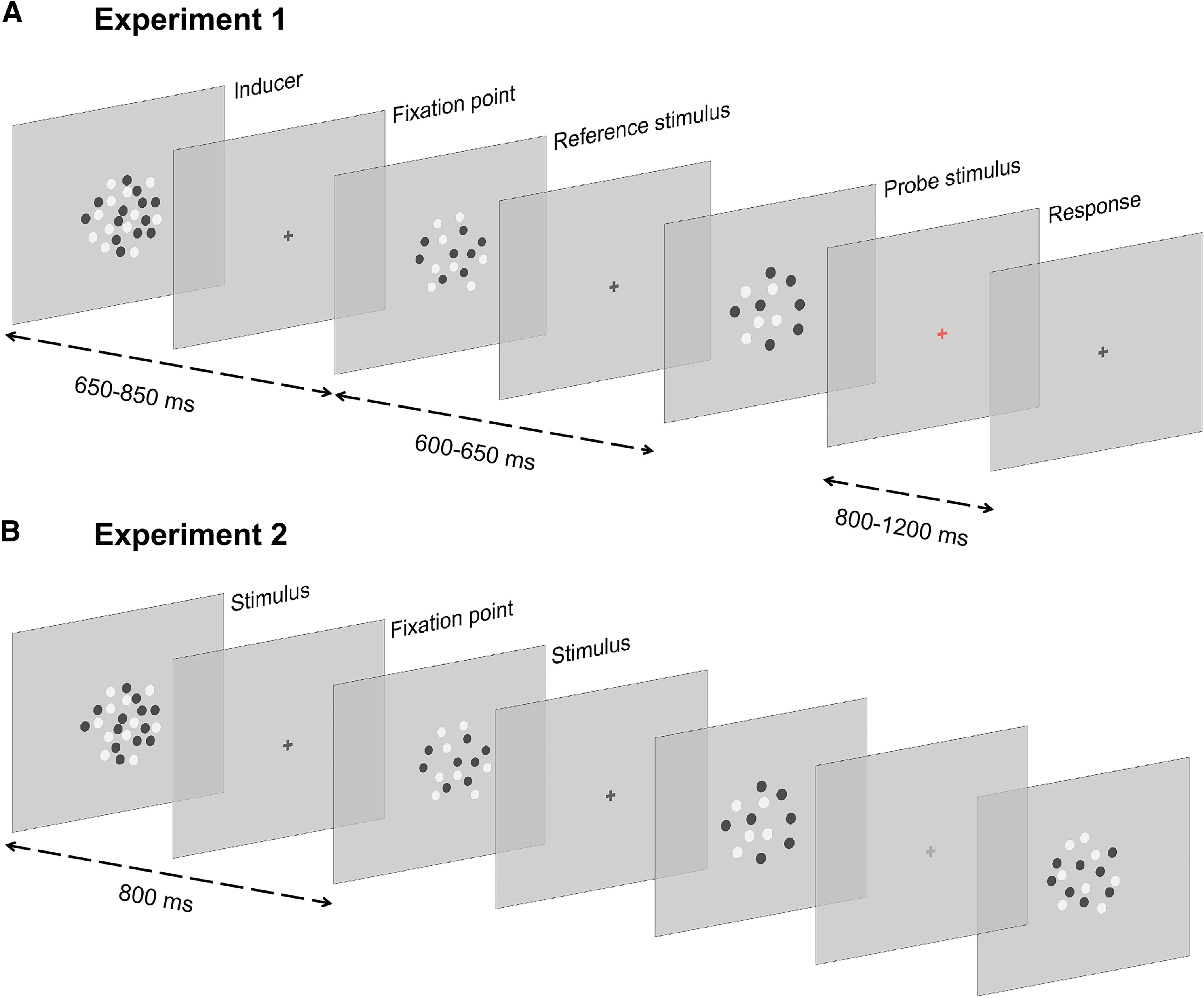
Experimental procedure. ***A***, Stimulation procedure used in Experiment 1. In Experiment 1, participants performed a numerosity, duration, or size discrimination task, in separate sessions. In each trial, we presented a sequence of three dot-array stimuli modulated in numerosity, duration, and dot size: a task-irrelevant “inducer” (either 12 or 24 dots, 140 or 280 ms, 4 or 8 pixels), a constant reference (always 16 dots/200 ms/6 pixels), and a variable probe (varying in numerosity, duration, or dot size according to the task). At the end of each trial, participants reported which stimulus between the reference and the probe contained more dots, lasted longer in time, or had bigger dots (for the numerosity, duration, and size task, respectively). ***B***, In Experiment 2, we used a passive viewing paradigm. Participants watched a stream of dot-array stimuli modulated in numerosity, duration, and dot size. Each stimulus, comprised of 12, 16, or 24 dots, was presented for 140, 200, or 280 ms, and included items with size of 4, 6, or 8 pixels. Participants were instructed to attend the sequence and respond to occasional oddball stimuli defined by a lower contrast. Stimuli are not depicted in scale.

In Experiment 1, we addressed the link between serial dependence and the neural signature of stimulus history across different magnitude dimensions and task conditions. The experiment (depicted in Fig. 1*A*) was divided into three different conditions (numerosity, duration, size), performed by participants (*N* = 30) in random order. In all the task conditions, participants discriminated the relevant magnitude (either numerosity, stimulus duration, or dot size) of a reference dot-array (always 16 dots, lasting 200 ms, and with dot size equal to 6 pixels) compared with a variable probe (ranging from 8-32 dots, from 100-400 ms, and from 3-12 pixel in the numerosity, duration, and size task, respectively). To induce serial dependence, a task-irrelevant inducer stimulus was presented before the reference, and was modulated across the three dimensions (with a numerosity of either 12 or 24 dots, a duration of 140 or 280 ms, and dot size of 4 or 8 pixels) in all the conditions.

**Figure 2.**
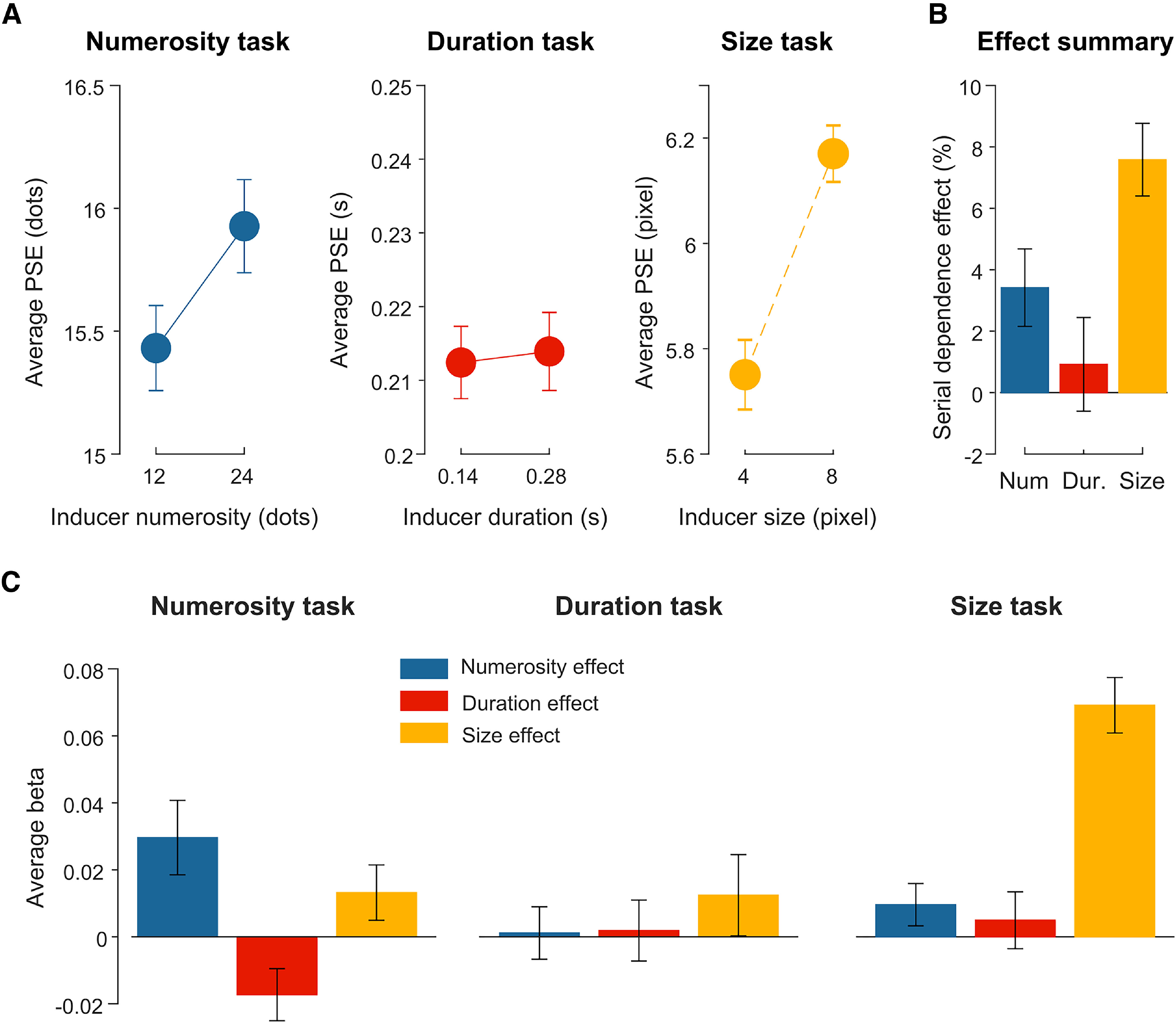
Behavioral results of Experiment 1. ***A***, Behavioral results in terms of point of subjective equality (PSE; i.e., representing accuracy in the task and the perceived magnitude of the reference) as a function of different inducer magnitudes, limited to the task-relevant magnitude. ***B***, Average serial dependence effect indexes computed as the normalized difference between PSEs in the two corresponding inducer conditions, transformed into percentage. ***C***, Effect of the different inducer magnitudes on behavioral performance, computed with a nonlinear regression analysis (i.e., contribution of different inducer magnitudes to the behavioral response in each trial). In this analysis, positive β values indicate an attractive effect (i.e., increased chance of responding “reference bigger” as the inducer magnitude increases), and negative results indicate a repulsive (opposite) effect. Error bars indicate SEM.

To assess the effect at the behavioral level, we first computed the PSE, a measure of the perceived magnitude of the reference stimulus (Fig. 2*A*) within each task condition and assessed how the perception of the reference stimulus is modulated by the corresponding inducer magnitude (i.e., numerosity in the numerosity task, and so on). To do so, we performed a series of paired *t* tests within each condition, controlling for multiple comparisons using an FDR procedure with *q* = 0.05. The *p* values reported thus reflect the *p* values adjusted by the FDR (adj-*p*). The results show a significant difference in PSE as a function of inducer magnitude in the numerosity (*t*_(29)_ = 2.60, adj-*p* = 0.022, Cohen's *d* = 0.48) and size (*t*_(29)_ = 7.79, adj-*p* = 0.003, Cohen's *d* = 1.45) task, suggesting that the higher the inducer magnitude (more numerous, bigger dot size), the higher the perceived magnitude of the reference. No effect was instead observed in the duration task (*t*_(29)_ = 0.47, adj-*p* = 0.64). To compare the effect across different conditions, we also computed a serial dependence effect index based on the normalized difference in PSE (Fig. 2*B*). On average, the serial dependence effect resulted as 3.42 ± 1.26%, 0.92 ± 1.53%, and 7.58 ± 1.18% for the numerosity, duration, and size task, respectively. The effect in the size task was significantly higher compared with the other two conditions (*t*_(29)_ = 2.56, adj-*p* = 0.016, *d* = 0.47, and *t*_(29)_ = 3.37, adj-*p* = 0.004, *d* = 0.61, respectively, for size vs numerosity and vs duration).

To further assess the serial dependence effect both within and across different dimensions, we performed a nonlinear regression analysis aimed to quantify the contribution of different inducer magnitudes to the discrimination judgment in each trial (Fig. 2*C*). The results showed a systematic influence of the inducer on perceptual judgments, in a task-specific fashion. Namely, we observed a significant effect of inducer numerosity in the numerosity task (β = 0.030 ± 0.060; one-sample *t* test against zero, *t*_(29)_ = 2.67, adj-*p* = 0.037, *d* = 0.5), and inducer size in the size task (β = 0.075 ± 0.033; *t*_(29)_ = 12.31, adj-*p* < 0.001, *d* = 2.3). Interestingly, in addition to the attractive (positive) effects, we also observed a repulsive (negative) effect provided by the inducer duration in the numerosity task (β = −0.017 ± 0.042), which, however, did not reach significance after controlling for multiple comparisons (*t*_(29)_ = −2.22, adj-*p* = 0.051, *d* = 0.4). This effect, however, showed a medium effect size (*d* = 0.4) similar to the effect of numerosity (*d* = 0.5), suggesting that it might reflect a genuine repulsive effect. No other significant contribution to behavioral responses was observed (all *t*_(29)_ ≤ 1.87, all adj-*p* ≥ 0.107).

Does the pattern of behavioral effects reflect differences in task difficulty? In terms of participants' precision in the task (Weber's fraction [WF]), we actually observed an opposite pattern of results compared with the effect. Indeed, the most difficult task resulted to be the duration task (i.e., highest Weber's fraction, WF [mean ± SEM] = 0.17 ± 0.017), followed by the numerosity task (WF = 0.12 ± 0.009), and finally the size task where precision was the highest (WF = 0.07 ± 0.003). A one-way repeated-measures ANOVA showed that there is indeed a significant difference between precision in the different tasks (*F*_(2,58)_ = 34.03, *p* < 0.001, η_p_^2^ = 0.281). The different difficulty of the three tasks is also reflected by different average response times, which were longest in the duration task (mean ± SEM = 498 ± 21 ms), again followed by the numerosity (378 ± 21 ms) and size task (358 ± 19 ms). There was a significant difference across response times in different tasks (one-way repeated-measures ANOVA, main effect of condition; *F*_(2,29)_ = 116.75, *p* < 0.001, η_p_^2^ = 0.80), with the duration task showing significantly slower responses compared with the other two conditions (paired *t* test; duration versus numerosity: *t*_(29)_ = 12.15, adj-*p* = 0.001; duration vs size, *t*_(29)_ = 14.10, adj-*p* = 0.001). This suggests that serial dependence in this context is unlikely to be associated with poorer performance, but it is instead strongest in the easiest task. Nevertheless, serial dependence might still be associated with poorer perceptual precision (Cicchini et al., 2018) within each task condition. A series of tests performed within each condition, however, did not show a consistent association between the serial dependence effect and the WF, neither in the numerosity (*r* = 0.09, *p* = 0.63) nor in the duration task (*r* = 0.31, *p* = 0.09). In the size task, we did observe a significant (negative) correlation, which was, however, driven by a single data point with particularly low WF (difference >2 SDs from the average). When excluding such data point, no significant correlation was observed (*r* = −0.04, *p* = 0.83).

**Figure 3.**
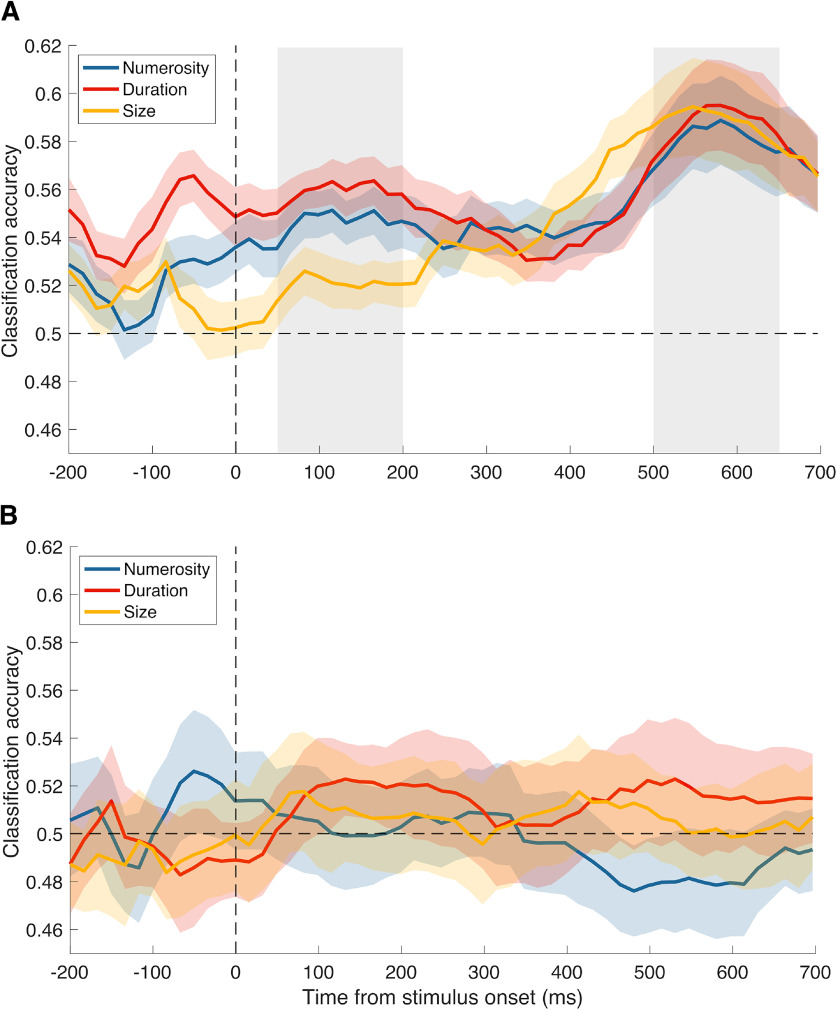
Average classification accuracy (CA) in Experiment 1. CA obtained in the multivariate analysis, showing the signature of the three magnitude dimensions of the inducer decoded from the EEG responses evoked by the reference, averaged across the three task conditions. The CA shown here reflects the ability of a classifier (support vector machine) to successfully classify the pattern of brain activity across multiple EEG channels evoked by the reference, according to the inducer magnitude (i.e., low vs high inducer numerosity, for example). Such procedure was repeated across several 100 ms time windows throughout an epoch (−200:700 ms) time-locked to the reference onset. ***A***, CA obtained in the actual decoding analysis. Gray shaded areas represent the two latency windows selected to perform further analyses (50-200 ms and 500-650 ms). ***B***, CA obtained in the “null” decoding analysis performed as a control and to determine the chance level empirically. Vertical dashed line indicates the onset of the reference stimulus. Horizontal dashed line indicates the level of 50% accuracy. Colored shaded areas represent the SEM.

To characterize the encoding of stimulus history (i.e., the magnitude information conveyed by the inducer) during the reference processing, we performed a multivariate pattern analysis in the time domain (e.g., King and Dehaene, 2014). The analysis involved the training and testing of a classifier (support vector machine) on EEG epochs time-locked to the reference stimulus, sorted according to the magnitude of the preceding inducer (i.e., low vs high numerosity, duration, or size). The resulting classification accuracy (CA) provides a measure of whether and to what extent the inducer magnitude is decodable from the brain responses to the reference stimulus. Figure 3*A* shows the average CA of the different task conditions, relative to the numerosity, duration, and size of the inducer. As shown in the figure, the decoding procedure yielded on average a good level of CA, showing both similarities (especially at late latencies, at ∼600 ms after stimulus) and differences (at earlier latencies) across the different conditions. In addition to our main decoding analysis, we also performed a control, “null” decoding analysis, in which the labels of the conditions being compared were shuffled before training the classifier. The classification accuracies obtained with this analysis (see Fig. 3*B*) were used to set the chance level empirically, and to control for spurious results.

**Figure 4.**
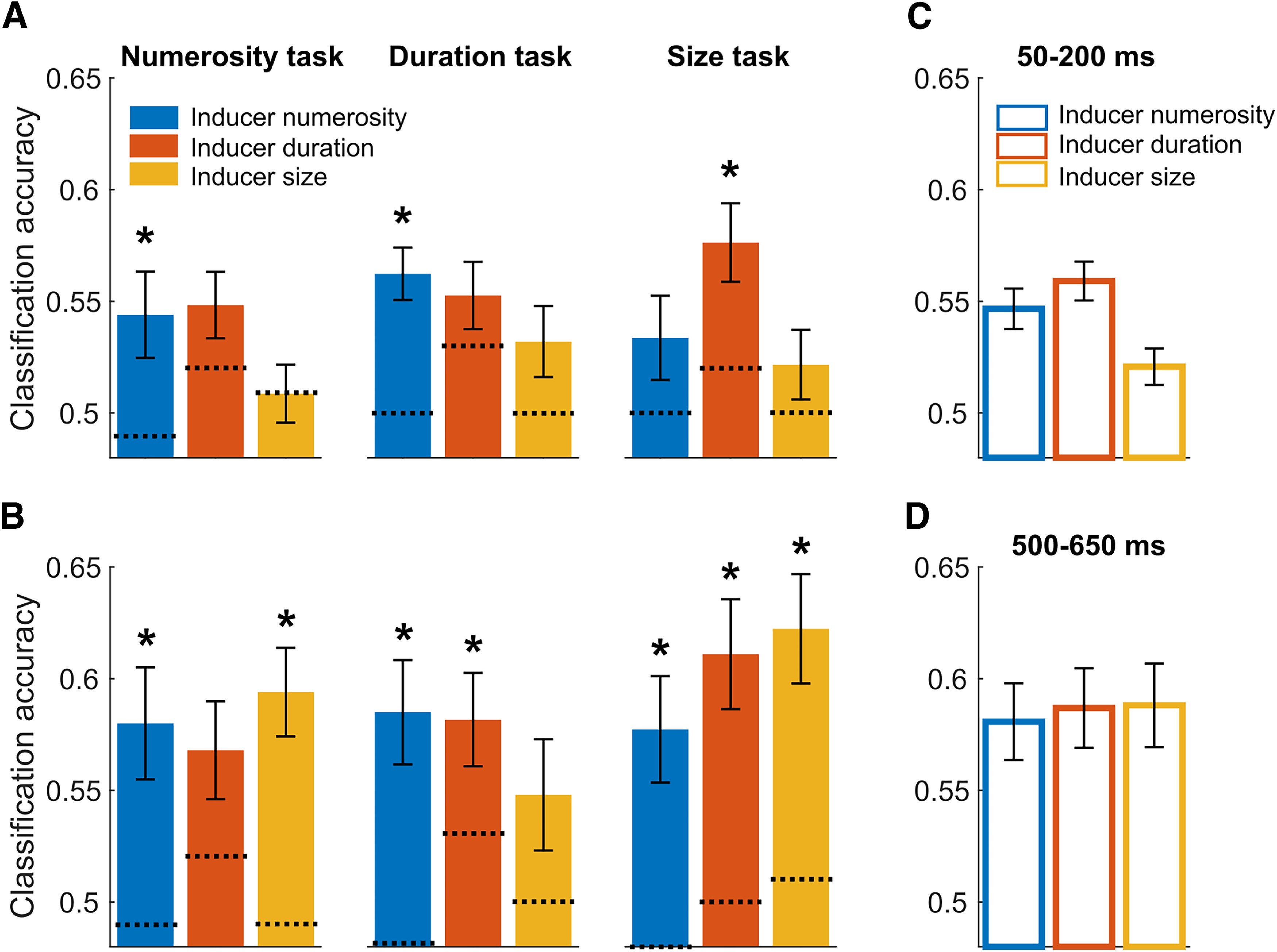
Decoding results of Experiment 1 at early and late latencies. ***A***, Average classification accuracies (CA) at the early latency window (50–200 ms), corresponding to the effect of inducer numerosity, duration, and size across the three task conditions (from the left to the right panel: numerosity, duration, and size task). ***B***, Average CA in the three task conditions at the late latency window (500-650 ms). The dotted line at each bar indicates the empirical chance level computed from the null decoding analysis. ***C***, CA corresponding to the effect of numerosity, duration, and size at the early latency window, averaged across the three tasks. ***D***, CA at the late latency window, averaged across the three tasks. Error bars indicate SEM. *p < 0.05.

To assess the neural signature of stimulus history and its pattern across magnitude dimensions and tasks, classification accuracies obtained throughout the reference epoch were averaged across two different time windows, spanning 50-200 ms and 500-650 ms (marked with shaded gray areas in Fig. 3*A*), separately for each inducer magnitude in each of the condition. Then, we checked for potential differences in the signature of the different inducer magnitudes across the three tasks. Figure 4*A*, *B* shows the average CA of the different inducer magnitudes for the three tasks and the two latency windows. First, to assess the pattern of results across the different conditions and dimensions, we performed a series of one-sample *t* tests against the corresponding average CA obtained in the null decoding analysis (marked in Fig. 4*A*,*B* with dotted lines). To control for multiple comparisons, we applied an FDR procedure within each time window (*q* = 0.05). In the early latency window, the effect of inducer numerosity was significantly higher than the (empirical) chance level in the numerosity (one-sample *t* test, *t*_(29)_ = 2.82, adj-*p* = 0.025) and in the duration (*t*_(29)_ = 4.83, adj-*p* < 0.001) task, but not in the size task (*t*_(29)_ = 1.54, adj-*p* = 0.201). The effect of inducer duration was significant only in the size task (*t*_(29)_ = 4.29, adj-*p* < 0.001), and not in the numerosity and duration task (*t*_(29)_ = 1.38-1.98, adj-*p* = 0.129-0.228). Finally, the effect of stimulus size was not significantly higher than chance level in any of the tasks (max *t*_(29)_ = −0.138 to 1.686, min adjusted *p* = 0.184). In the late latency window, with the exception of duration in the numerosity task (*t*_(29)_ = 1.38, adj-*p* = 0.064) and size in the duration task (*t*_(29)_ = 1.96, adj-*p* = 0.077), all the classification accuracies were significantly above the respective chance level measured empirically (*t*_(29)_ = 3.10-5.01, adj-*p* ≤ 0.025). These results suggest that the stimulus history information encoded in brain responses does not necessarily match the pattern of behavioral serial dependence effects as, for instance, we observed a significant decoding even for dimensions that did not significantly affect behavior.

We then performed a three-way repeated-measures ANOVA on the average CA computed in the two latency windows and in the different tasks, with factors “task” (i.e., numerosity, duration, and size task), “inducer magnitude” (i.e., inducer numerosity, duration, and size), and “latency window” (i.e., early vs late). This analysis showed no main effect of task (*F*_(2,58)_ = 1.093, *p* = 0.342), no main effect of inducer magnitude (*F*_(2,58)_ = 1.739, *p* = 0.185), but a significant main effect of latency window (*F*_(1,29)_ = 6.927, *p* = 0.013, η_p_^2^ = 0.193) and a significant interaction between inducer magnitude and latency window (*F*_(2,58)_ = 3.894, *p* = 0.026, η_p_^2^ = 0.119). No other interaction effect was observed (max *F* = 1.577, min *p* = 0.185).

To better understand the nature of this interaction, we followed it up by focusing on the average effect of different magnitudes at different time windows. Namely, as the task did not seem to significantly modulate the pattern of CA or interact with the other factors, we collapsed (averaged) the classification accuracies across the different tasks (Fig. 4*C*,*D*). Two separate one-way repeated-measures ANOVAs (with factor “inducer magnitude”) showed a significant main effect of inducer magnitude (*F*_(2,58)_ = 5.654, *p* = 0.006, η_p_^2^ = 0.163) in the early latency window (50-200 ms; Fig. 4*C*), and no significant difference across the inducer magnitudes (*F*_(2,58)_ = 0.168, *p* = 0.846) in the late latency window (500-650 ms; Fig. 4*D*). This difference in the pattern of decoding across the two latency windows thus explains the interaction observed in the previous test, and shows that, while the level of CA is more variable across magnitudes at early latencies, it becomes very similar at later latencies.

Finally, a paired *t* test comparing the average decoding performance in the early versus late latency window (i.e., average of the three bars in Fig. 4*C* vs the average of Fig. 4*D*) showed that classification accuracies were overall significantly higher at the late window (0.542 ± 0.005 vs 0.585 ± 0.016; *t*_(29)_ = 2.630, *p* = 0.0135, Cohen's *d* = 0.56). This suggests that the initial pattern of activity encoding the inducer magnitude information is amplified (i.e., stronger activity, or sharper representation) at later latencies.

**Figure 5.**
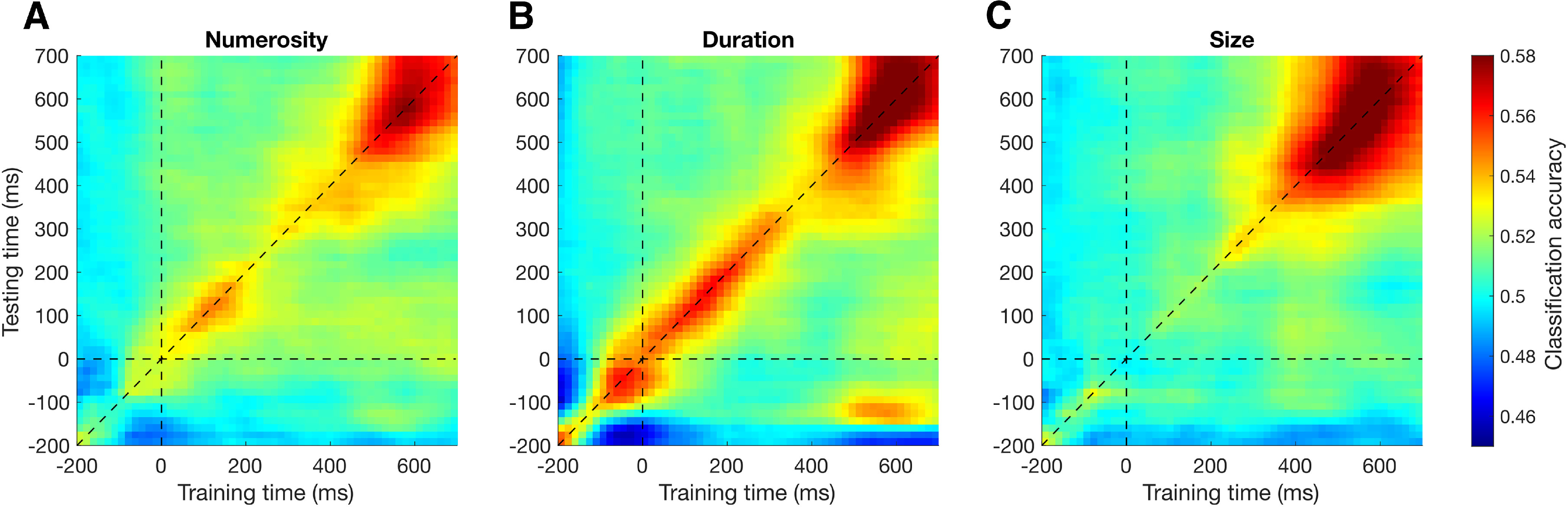
Average temporal generalization matrices in Experiment 1. The temporal generalization matrices are obtained by training and testing the classifier with brain activity at different latencies, to show the extent to which a given pattern of brain activity generalizes to later latencies. ***A***, Temporal generalization matrix concerning the effect of inducer numerosity, averaged across task conditions. ***B***, Temporal generalization matrix concerning the effect of duration. ***C***, Temporal generalization matrix concerning the effect of size. Horizontal and vertical dashed lines indicate the off-diagonal generalization direction corresponding to the reference onset. The diagonal dashed line indicates the training and testing of the classifier at the same latency. The classification accuracies shown in Figure 3*A* correspond to the diagonal of the temporal generalization matrices.

In our interpretation, the results of the decoding analysis show a signature of stimulus history affecting the processing of the reference. However, in the analysis, we also observed relatively high classification accuracies even before the onset of the reference stimulus, especially considering the duration condition (Figs. 3*A*, 5*B*). This raises the possibility that what we are measuring may not be a signature of the effect of stimulus history on the reference representation, but a lingering trace of the inducer stimulus itself. To address this possibility, we checked the temporal generalization matrices of each inducer magnitude effect, averaging the different tasks together (Fig. 5). The temporal generalization matrices are obtained by training and testing the classifier with brain activity at different latencies, to assess whether specific patterns of activity generalize to different latencies. Borrowing from the interpretations provided by King and Dehaene (2014), a lingering trace of the inducer stimulus is expected to emerge in the temporal generalization matrices as a relatively stable signature encompassing the prestimulus interval and extending to poststimulus latencies. In the case of numerosity (Fig. 5*A*) and size (Fig. 5*C*), we observed relatively weak activity in the prestimulus interval, which instead starts to rise only after the onset of the reference stimulus. In line with the plots shown in Figure 3*A*, which represent the diagonals of the temporal generalization matrices, numerosity shows an early peak at ∼150 ms after stimulus followed by a later peak at ∼600 ms, while size only shows a main peak at late latencies. In these two cases, there is no evidence of early decoding capturing a lingering trace of the inducer stimulus. In the case of duration (Fig. 5*B*), prestimulus classification accuracies appeared to be stronger. However, the decoding showed a pattern mostly unfolding along the diagonal, with little generalization. This suggests the presence of distinct patterns of brain activity evolving over time (see King and Dehaene, 2014), which are more in line with an active processing of stimulus history rather than with a passive trace of the inducer stimulus. Additionally, in all three cases, the large peak observed toward the end of the stimulus epoch is not consistent with a trace of the past stimulus, which would instead be expected to decay over time. Overall, the temporal generalization plots provided little evidence for the presence of a lingering trace of the inducer stimulus. More likely, the prestimulus decoding could represent an anticipatory response to the reference because of the relatively narrow jittering of its onset, or a by-product of the sliding window average used in our decoding analysis. Although we cannot conclusively exclude the presence of a lingering trace of the inducer stimulus, the specific patterns of decoding observed in the analysis suggest that, if present, such a trace would likely interact with the processing of the reference stimulus.

In addition to the neural signature of stimulus history per se, an important question is whether such a signature relates to the attractive effect observed behaviorally. Therefore, to establish a link between the behavioral and neural effect, we addressed whether the strength of the serial dependence effect reflects the extent to which past information is encoded in brain signals, as indicated by the CA. To do so, we performed a series of LME model tests aimed at assessing whether the behavioral effect (i.e., normalized difference between PSEs obtained as a function of different inducer magnitudes; see Fig. 2*B*) could be predicted by the CA obtained in the decoding analysis. In these tests, the behavioral effect thus represented the dependent variable, the CA the fixed effect, and the subjects were added as the random effect (Eff ∼ CA + (1|subj)). This analysis was performed separately in the different task conditions and the two latency windows used in the previous analysis (50-200 ms, 500-650 ms). The results showed that the strength of the behavioral effect can be successfully predicted by the CA in the early latency window (50-200 ms) when considering the effect of numerosity in the numerosity task and the decoding of inducer numerosity (*R*^2^ = 0.68, *t* = 2.69, *p* = 0.006), and the effect of size in the size task and the decoding of inducer size (*R*^2^ = 0.66, *t* = 2.64, *p* = 0.013). No other test reached significance in the early latency window (*t* spanning from −1.08 to 0.60, min *p* = 0.29). In the late latency window, no significant relationship between the behavioral effect and the CA was observed (*t* spanning from −1.56 to 1.32, min *p* = 0.13).

**Figure 6.**
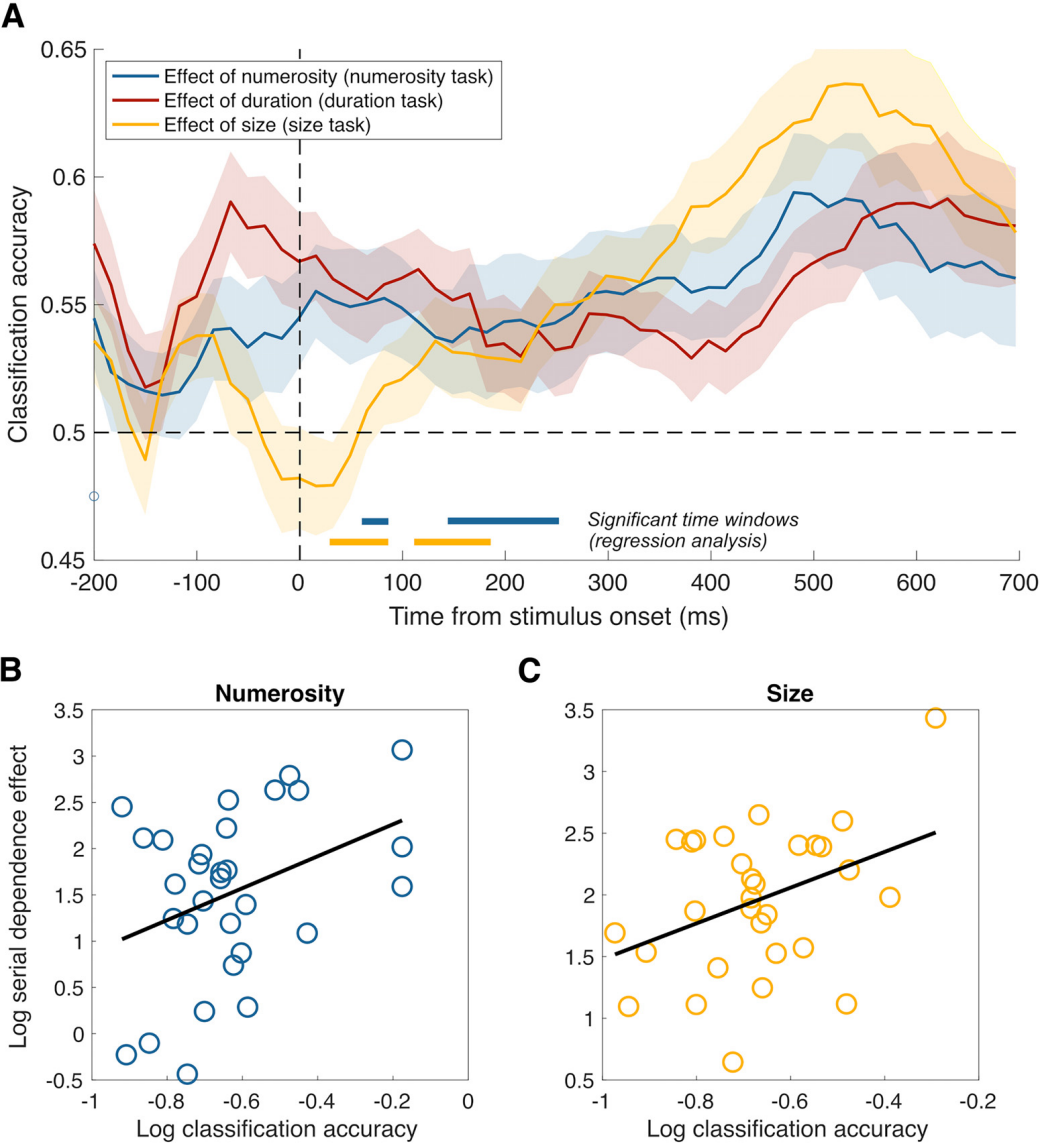
Classification accuracies (CA) of the task-relevant dimensions in the three task conditions. ***A***, Pattern of CA obtained from the decoding of the magnitude dimension of the inducer consistent with the task performed, in the three task conditions. Lines at the bottom of the plot indicate the significant time windows observed in the regression analysis, showing the latency windows at which the behavioral effect could be predicted from CA. Shaded areas represent SEM. ***B***, Log-scaled CA plotted against the serial dependence effect, in the numerosity task condition. ***C***, Log-scaled CA plotted against the serial dependence effect, in the size task condition. Black lines indicate linear fit to the data.

In addition to these tests focused on the two large latency windows used in the previous analyses, we also took a more comprehensive approach and performed a series of LME tests at different latencies throughout the reference epoch (−200:700 ms), to assess whether the strength of the behavioral effect could be predicted by the CA. To limit the number of tests, we only considered the classification accuracies and behavioral effects of the task-relevant magnitude in each task condition (i.e., effect of numerosity in the numerosity task, duration in the duration task, size in the size task). The pattern of classification accuracies corresponding to this subset of conditions is shown in Figure 6. In this analysis, we considered an effect significant only when showing at least two consecutive significant time windows, and applied a cluster-based nonparametric test to control for multiple comparisons (see below). In the numerosity task condition, the CA relative to the inducer numerosity significantly predicted the behavioral effect provided by the inducer numerosity across two clusters: a smaller one spanning from 65 to 85 ms after stimulus onset (2 consecutive time windows; average *R*^2^ = 0.62, *t* values = 2.15-2.17, *p* = 0.038-0.041), and a larger one from 145 to 245 ms (7 time windows; average *R*^2^ = 0.65, *t* values = 2.10-2.86, *p* = 0.008-0.045). In the duration task, no significant effect was observed (max *t* = 1.82, min *p* = 0.08). Finally, in the size task condition, we observed again two clusters of significant effects showing a relationship between the behavioral effect and the CA, one spanning from 35 to 85 ms (4 time windows; average *R*^2^ = 0.63, *t* values = 2.06–2.57, *p* = 0.015-0.048), and another one going from 115 to 180 ms (5 time windows; average *R*^2^ = 0.65, *t* values = 2.13-2.96, *p* = 0.006-0.042). The significant clusters are marked in Figure 6*A* at the bottom of the plot, with the same color code as the main plots. To ensure the robustness of these clusters of significant effects, we also performed a cluster-based nonparametric test, shuffling the data entered into the LME test and assessing the proportion of times that a similar cluster could be observed by chance (10,000 repetitions for each cluster). The cluster-level *p* value obtained in this way was in all the cases < 0.001. Compared with the broad latency windows used in the previous analysis, these tests allowed to identify with greater temporal precision the early latencies at which a relationship between behavioral effect and neural signature of stimulus history emerges.

To show the direction of the relationship captured with the regression analysis, we also plotted the (log-scaled) average CA observed across the significant time windows against the behavioral effect (limited to numerosity and size; Fig. 6*B*,*C*). Both plots show that there is a positive relationship between these two measures, with stronger behavioral effects associated with higher classification accuracies. In both cases, we observed a significant correlation between the two measures (*r* = 0.37, *p* = 0.043 and *r* = 0.39, *p* = 0.035, respectively, for numerosity and size).

Note that the significance of the regression analysis does not depend on the absolute value of the CA, but on the pattern across the group as a function of the behavioral effect. In the size condition, indeed, a significant relationship between CA and behavioral effect was observed at latencies that show weak decoding, close to the 50% probability level. Despite the weak decoding, differences across the group can still show a relationship with the strength of the behavioral effect.

### Experiment 2

The results of Experiment 1 showed that (1) while the behavioral serial dependence effect shows different patterns according to the task performed by participants, with task-specific effects, the decoding of stimulus history shows a more generalized signature also reflecting inducer's magnitudes that did not yield a behavioral effect; and (2) we nevertheless observed a link between the behavioral effect and its neural signature, emerging at early latencies after stimulus onset.

In Experiment 2, we further asked whether the neural signature of stimulus history might be modulated by task-related factors. Previous results show that stimulus history can be decoded from neural signals in a passive viewing paradigm (Fornaciai and Park, 2018a), suggesting that the encoding and implementation of stimulus history does not hinge on the presence of an active task. However, it is unclear whether the signature of stimulus history would be completely independent from what the participants are doing, or whether its strength could be modulated by being engaged in a task. Our aim was thus to compare the strength and pattern of stimulus history decoding with the results of Experiment 1, to address the potential modulatory role of the task as opposed to a passive viewing of a series of stimuli.

To address this question, we used a passive viewing paradigm, keeping the stimulation procedure as similar as possible to Experiment 1. In this experiment, participants (*N* = 29) observed a sequence of dot-array stimuli varying in numerosity, duration, and dot size (Fig. 1*B*), and responded to occasional odd-ball stimuli defined by a lower contrast (i.e., to ensure that participants kept watching and attending the stimuli). We then used again a multivariate decoding procedure to assess the encoding of past stimulus information in visual-evoked activity. Since every stimulus in the sequence varied along the three dimensions (i.e., instead of having a constant reference), the sorting of trials used to perform the analysis differed from Experiment 1. Namely, in different iteration of the analysis, we took all the trials in which the intermediate magnitude level of each dimension was presented (i.e., 16 dots, 200 ms, or 6 pixels, henceforth called “current magnitude”) and assessed the effect of different magnitude dimensions of the stimulus presented in the preceding trial. The decoding was thus performed on EEG activity time-locked to an identical “current” stimulus with the intermediate magnitude level (equivalent to the reference stimulus in Experiment 1), as a function of the magnitude of the preceding one. In different iterations of the analysis, we thus compared cases where the past stimulus had 12 versus 24 dots, a duration of 140 versus 280 ms, or a dot size of 4 versus 8 pixels (for numerosity, duration, and size, respectively; henceforth called “past magnitude”).

**Figure 7.**
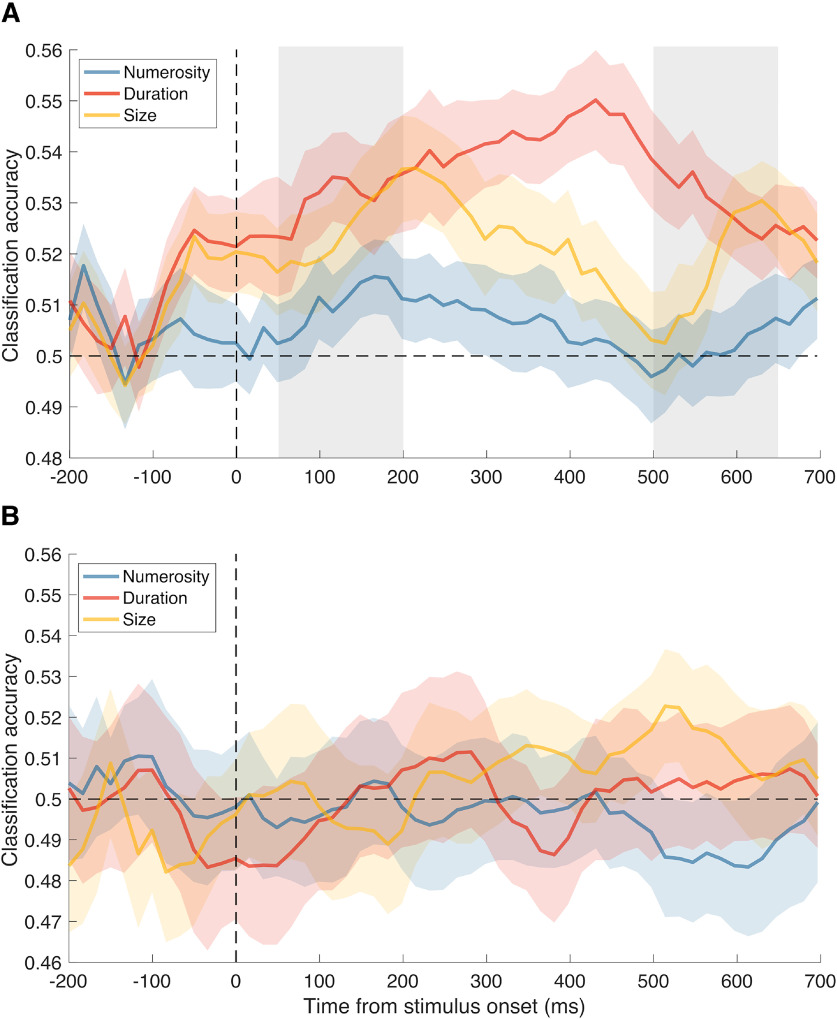
Average classification accuracy (CA) in Experiment 2. In Experiment 2, participants passively watched a stream of dot-array stimuli that were concurrently modulated in numerosity, duration, and dot size in a trial-by-trial fashion. In the multivariate decoding procedure, in different iterations of the analysis, we pooled all the stimuli with the intermediate level of either numerosity, duration, or dot size (named “current” magnitude in the description of the results), and decoded the signature of the preceding stimulus by contrasting trials in which the previous stimulus had a lower magnitude (i.e., either 12 dots, 140 ms, or 4 pixels, to assess the effect of numerosity, duration, or dot size, respectively; named “past” magnitude) against trials in which the previous stimulus had a larger magnitude (i.e., 24 dots, 280 ms, or 8 pixels). The decoding was thus performed by considering the activity time-locked to an identical stimulus with an intermediate magnitude level, differing only in the magnitude of the preceding stimulus, similarly to the procedure used in Experiment 1. ***A***, CA observed in the actual decoding analysis, corresponding to the different magnitudes of the past stimulus. Two gray shaded areas represent the latency windows selected to perform data analysis, as in Experiment 1. ***B***, CA obtained in the null decoding analysis, serving as a control and to determine the chance level empirically. Vertical dashed lines indicate the onset of the reference stimulus. Horizontal dashed lines indicate the level of 50% accuracy. Colored shaded areas represent the SEM.

**Figure 8.**
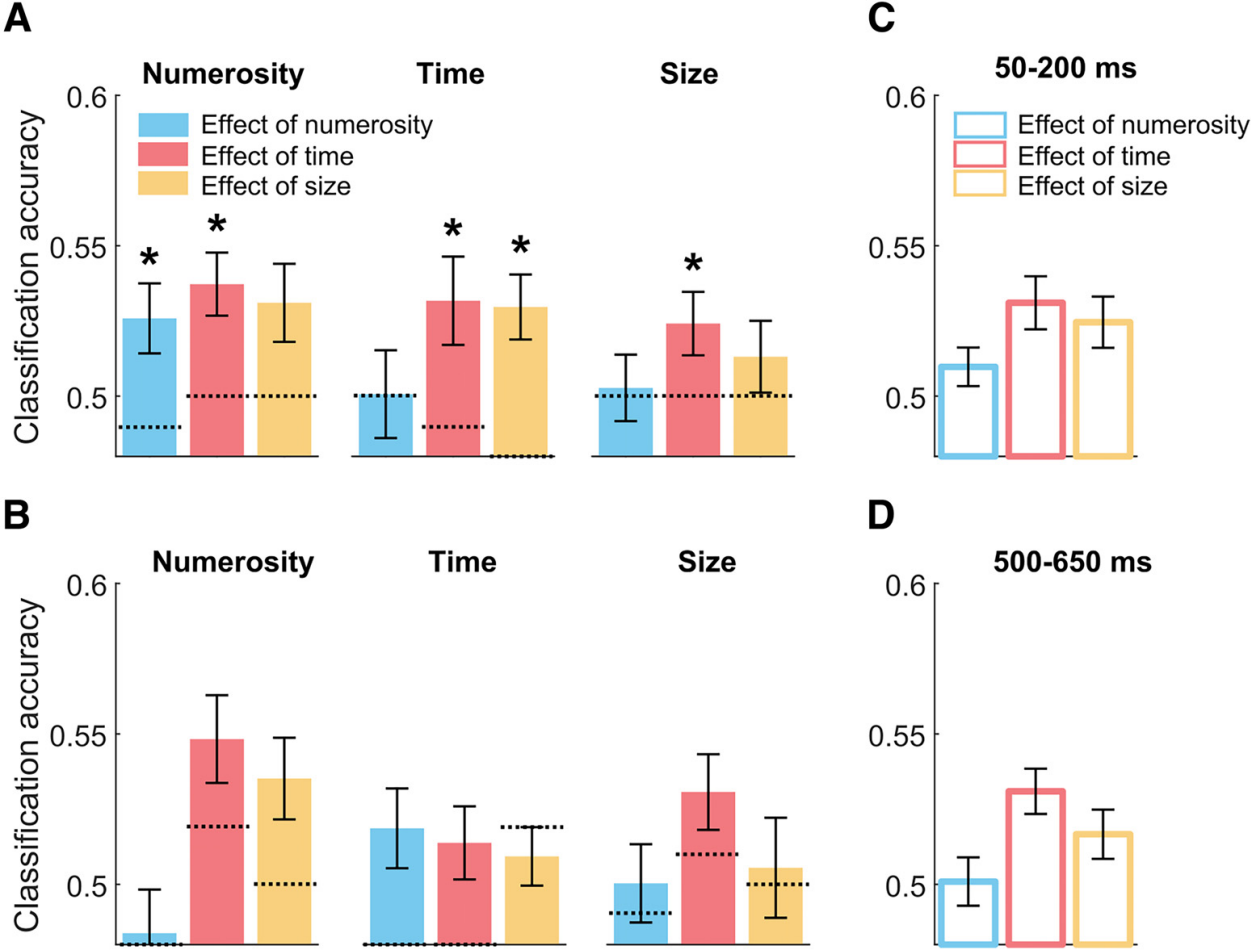
Decoding results of Experiment 2 at early and late latencies. ***A***, ***B***, For each magnitude dimension of the current stimulus (i.e., “current magnitude,” different subplots), we plotted the classification accuracy (CA) of the “past” magnitude in the early (***A***, 50-200 ms) and late (***B***, 500-650 ms) latency window. Namely, from left to right, bars represent the CA corresponding to the effect of numerosity (blue), duration (red), and size (yellow) of the past stimulus on the numerosity (leftmost panel), duration (middle panel), and size of the current stimulus (rightmost panel). Dotted lines shown at each bar indicate the empirical chance level computed from the null decoding analysis. ***C***, Average effect of the magnitudes of the previous stimulus on the current one in the early latency window, collapsing together the different magnitudes of the current stimulus. ***D***, Average effects of different magnitudes in the late latency window. Error bars indicate SEM. *p < 0.05.

Figure 7*A* shows the average classification accuracies relative to the decoding of different numerosities, durations, or dot sizes of the preceding stimulus, while Figure 7*B* shows the results of the null decoding analysis performed with shuffled data. Overall, although generally weaker compared with Experiment 1, the analysis was able to capture the signature of the past stimulus also in this case, while the null decoding analysis did not show any consistent decoding. To better assess the signatures of different magnitude dimensions, we again computed the average CA in two separate latency windows (early window: 50-200 ms; late window: 500-650 ms; marked with shaded areas in Fig. 7*A*). The classification accuracies observed within these two latency windows, corresponding to the different combinations of current and past magnitude, are shown in Figure 8*A*, *B*. Each subpanel in Figure 8*A*, *B* refers to the “current” magnitude, while each bar refers to the decoding accuracy of the “past” magnitude. For example, the first subpanel in Figure 8*A* shows the effect of numerosity (12 vs 24 dots; blue bar), duration (140 vs 280 ms; red bar), and dot size (4 vs 8 pixels; yellow bar) on stimuli having a numerosity of 16 dots (i.e., the intermediate numerosity of the range). Figure 8*C*, *D* shows instead the effect of the different “past” magnitude regardless of the “current” magnitude (the magnitude of the current stimulus). Figure 8*C*, *D* indeed shows the average of the three subpanels of Figure 8*A* and 8*B*, respectively.

To assess the pattern of decoding across different magnitudes, we first performed a series of one-sample *t* tests against the corresponding average CA observed in the null decoding analysis. Additionally, we applied FDR to control for multiple comparisons (*q* = 0.05). In the early latency window (Fig. 8*A*), the results showed that the decoding of numerosity was significantly above chance only when assessing the effect on numerosity in the current trial (*t*_(28)_ = 3.30, adj-*p* = 0.008; other tests: *t*_(28)_ = −0.20-0.003, min adj-*p* = 0.943). Duration was instead significant in all cases (*t*_(28)_ = 2.47-3.92, adj-*p* < 0.036). Finally, the decoding of size showed a significantly above chance accuracy only when considering the effect of size on duration (*t*_(28)_ = 4.48, adj-*p* = 0.001; other tests: *t*_(28)_ = 0.84-2.18, min adj-*p* = 0.057). In the late latency window (Fig. 8*B*), instead, we did not observe any significant CA after correction for multiple comparisons (*t*_(28)_ = −1.78-2.60, min adj-*p* = 0.069).

Similarly to Experiment 1, we then performed a three-way repeated-measures ANOVA, with factors “current magnitude” (i.e., numerosity, duration, size), “past magnitude,” and “latency window.” The results showed no main effect of current magnitude (*F*_(2,56)_ = 1.613, *p* = 0.208), no main effect of latency window (*F*_(1,28)_ = 0.950, *p* = 0.338), but a significant main effect of past magnitude (*F*_(2,56)_ = 5.163, *p* = 0.006, η_p_^2^ = 0.155). More importantly, we observed a significant three-way interaction (*F*_(4112)_ = 2.868, *p* = 0.026, η_p_^2^ = 0.094) between the current magnitude, past magnitude, and latency window.

To further explore this three-way interaction, we assessed the pattern of effects across different combinations of current and past stimulus magnitude separately at early (Fig. 8*A*) and late (Fig. 8*B*) latencies, performing two independent two-way ANOVAs with factor “current magnitude” and “past magnitude.” In the early latency window, we observed no main effect of either current and past magnitude, and no interaction between the two factors (max *F* = 2.533, min *p* = 0.089), suggesting that, although the decoding of numerosity (Fig. 8*A*, blue bars) appears to be weaker than the decoding of the other magnitudes (especially when considering duration and size as current magnitudes; Fig. 8*A*, middle and leftmost panels), the overall difference is not large enough to reach significance. In the late latency window (Fig. 8*B*) instead, we observed a significant main effect of the “past” magnitude (*F*_(2,56)_ = 3.611, *p* = 0.030, η_p_^2^ = 0.113), no main effect of the “current” magnitude, and no interaction (max *F* = 2.245, min *p* = 0.069), suggesting a greater difference in the decoding accuracy between different past magnitudes. Thus, differently from the results of Experiment 1 (where we found that classification accuracies were more variable at early latencies), here there was a greater difference in the decoding accuracy between magnitudes at later latencies.

**Figure 9.**
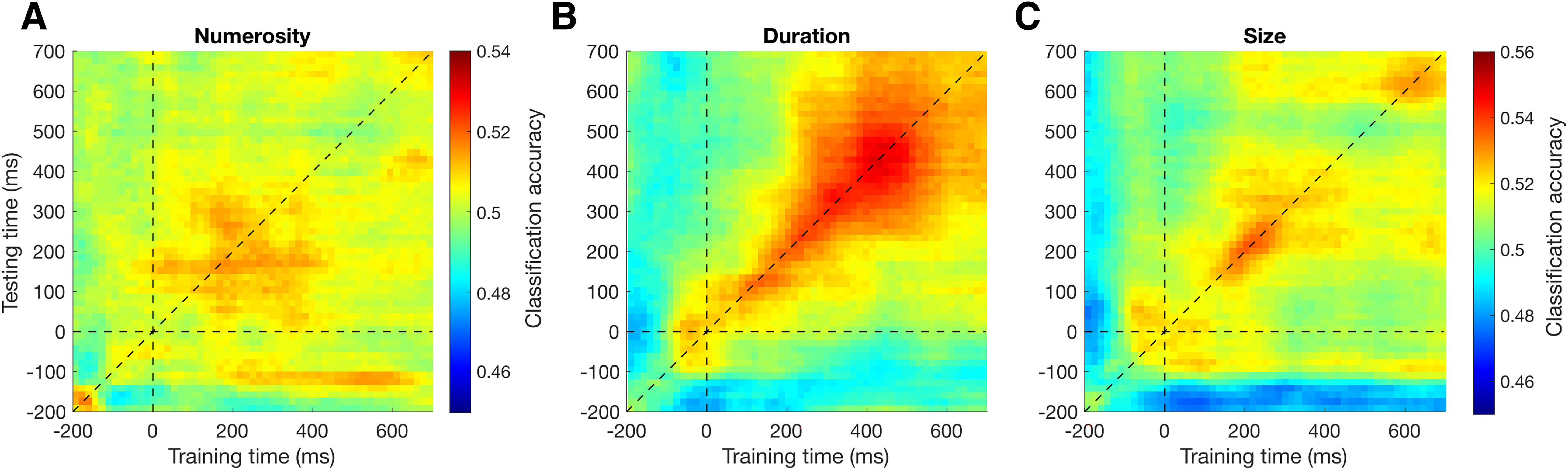
Temporal generalization matrices of Experiment 2. ***A***, Temporal generalization matrix concerning the effect of the numerosity of the past stimulus. ***B***, Temporal generalization matrix concerning the effect of duration. ***C***, Temporal generalization matrix concerning the effect of size. Horizontal and vertical dashed lines indicate the off-diagonal generalization direction corresponding to the current stimulus onset. The diagonal dashed line indicates the training and testing of the classifier at the same latency. The classification accuracies shown in Figure 7*A* correspond to the diagonal of the temporal generalization matrices.

Since classification accuracies seemed again to increase above chance level before the stimulus onset, we assessed the temporal generalization matrices (Fig. 9) to better understand the nature of this effect, similarly to Experiment 1. In all three cases (concerning the different magnitude dimensions), the temporal generalization provided little evidence for the presence of a lingering trace of the previous stimulus. Indeed, although the classification accuracies started increasing during the prestimulus interval, the temporal generalization did not show any relatively stable pattern independent from the onset of the current stimulus. In all cases, the CA peaked after the onset of the current stimulus, suggesting an interaction between stimulus history and the processing of the current stimulus. As in Experiment 1, the pattern of classification accuracies before the onset of the current stimulus might represent either an anticipatory activation or a by-product of the sliding window average used in the decoding analysis. If present, any lingering trace of the past stimulus would most likely interact with the processing of the current stimulus, showing an increase in CA at poststimulus latencies rather than a decay.

### Comparison between Experiments 1 and 2

Finally, we directly compared the results of Experiments 1 and 2, to assess the influence of task context on the neural signature of stimulus history. To do so, we used a mixed-model ANOVA, with “reference magnitude” (i.e., coding for the different task conditions in Experiment 1, and for the different “current magnitudes” in Experiment 2), “past magnitude” (i.e., inducer magnitude in Experiment 1, and magnitudes of the previous trial in Experiment 2), and “latency window” (early vs late) as within-subject factors, and “experiment” (Experiment 1 vs Experiment 2) as between-subject factor. The results showed a main effect of past magnitude (*F*_(2194)_ = 4.192, *p* = 0.018, η_p_^2^ = 0.068), a main effect of latency window (*F*_(1,57)_ = 4.530, *p* = 0.038, η_p_^2^ = 0.073), and a main effect of experiment (*F*_(1,57)_ = 21.723, *p* < 0.001, η_p_^2^ = 0.276). We also observed a significant interaction between latency window and experiment (*F*_(1,57)_ = 7.665, *p* = 0.008, η_p_^2^ = 0.118), suggesting that the signature of stimulus history at different latencies depends on whether the experiment involves an engaging magnitude task, or a passive viewing of the visual stimuli. No other main effect or interaction reached significance (max *F* = 2.289, min *p* = 0.061).

**Figure 10.**
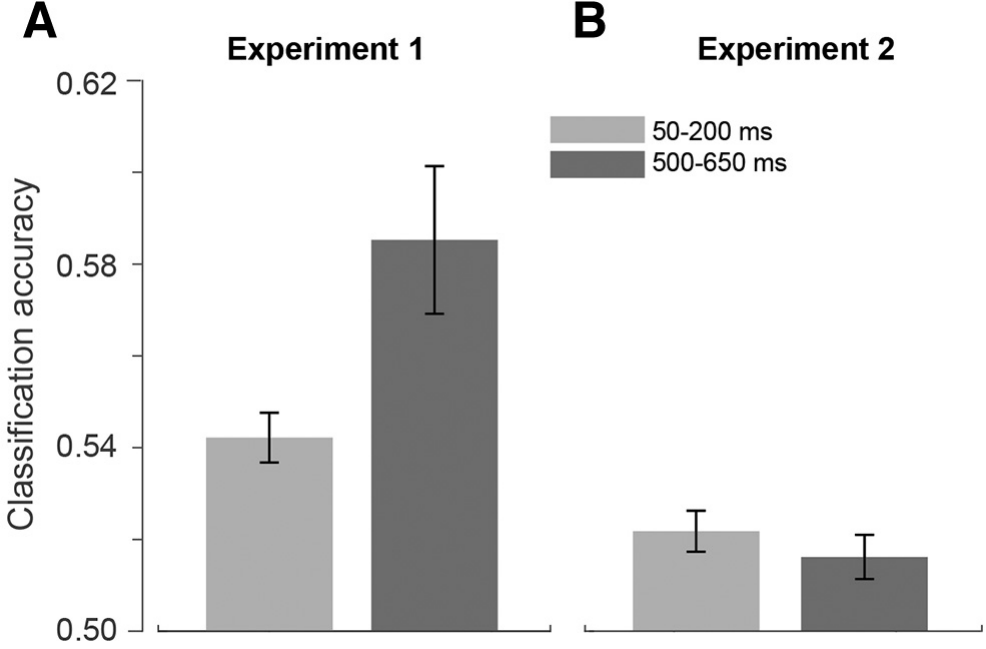
Comparison between Experiments 1 and 2. ***A***, Average classification accuracy (CA) across all the conditions and dimensions tested in Experiment 1. The results of Experiment 1 show that, while a signature of the inducer magnitude information is on average measurable from neural signals very early after the reference stimulus onset (50-200 ms), such a signature is strongly amplified at later latencies (500-650 ms). ***B***, Average CA obtained in Experiment 2. Although significantly higher than chance level (0.5), in Experiment 2 the decoding performance shows an overall weaker encoding (lower CA) of the inducer magnitudes during reference processing, compared with Experiment 1. At the late latency window, no amplification of these signals was observed. Error bars indicate SEM.

We further performed a series of *post hoc* tests assessing the average CA in the two temporal windows separately for each experiment (Fig. 10). First, a series of one-sample *t* tests against chance level (i.e., average CA of the null decoding analysis), corrected with FDR, showed that in all cases CA was significantly higher than chance level (all *t* values ≥ 3.15, all adj-*p* ≤ 0.004). Then we performed a paired *t* test (corrected with FDR) comparing the CA at different temporal latencies within each experiment. The results showed that while in Experiment 1 there was a significant difference between temporal windows, with higher CA at later temporal latencies (*t*_(29)_ = 2.63, adj-*p* = 0.026, *d* = 0.56), there was no significant difference between latencies in Experiment 2 (*t*_(28)_ = 0.953, adj-*p* = 0.35). Furthermore, we averaged together classification accuracies corresponding to different latency windows within each experiment, and compared the two experiments against each other. This test showed that CAs were on average significantly higher in Experiment 1 compared with Experiment 2 (0.56 ± 0.009 vs 0.52 ± 0.003, respectively; independent-sample *t* test, *t*_(57)_ = 4.66, *p* < 0.001, *d* = 1.25).

## Discussion

In this study, we addressed the link between behavioral serial dependencies observed in perceptual tasks (Fischer and Whitney, 2014), and the neural signature of stimulus history emerging from visual-evoked potentials (Fornaciai and Park, 2018a). In doing so, we had two main goals: (1) understanding whether the neural signature of stimulus history is a direct correlate of the behavioral effect and (2) pinpoint the brain processing stages linked to the emergence of the behavioral bias. The nature of serial dependence is indeed debated, and interpretations on the origin of this effect span from basic sensory/perceptual processes (Burr and Cicchini, 2014; Fischer and Whitney, 2014; Fornaciai and Park, 2018a) to high-level processes related to memory or decision-making (Bliss et al., 2017; Fritsche et al., 2017; Pascucci et al., 2019). Our study first demonstrates that, while serial dependence is selective for the task at hand, the neural signature of stimulus history encompasses multiple dimensions of the past stimulus, including dimensions that do not yield a behavioral effect. Second, we observed a relationship between the strength of the behavioral effect and the decoding accuracy of past stimulus information. Third, the encoding of past information is weaker during passive viewing, suggesting that attention or task relevance modulates the extent to which stimulus history is encoded in brain signals.

Regarding the behavioral results, our data highlight three main features of serial dependence in magnitude perception. First, consistently with previous results (Van der Burg et al., 2019; Togoli et al., 2021), the effect appears to be specific for the task. This in turn suggests that attention or other factors like the task set likely play a role in determining the effect when different dimensions are simultaneously manipulated, in line with the idea that serial dependence depends on attention (Fischer and Whitney, 2014; Fornaciai and Park, 2018a; Collins, 2019; Fritsche and de Lange, 2019; Manassi and Whitney, 2022). Second, we observed a repulsive effect, akin to perceptual adaptation (Kohn, 2007), provided by the inducer duration in the numerosity task. Although not statistically significant after correcting for multiple comparisons, this effect still showed a medium effect size, and is in line with previous results (Togoli et al., 2021). Third, duration perception seems in this context free from serial dependence, although previous results showed significant effects in a similar task (Togoli et al., 2021). However, this is likely explained by the small range of inducer durations, making the inducer too similar to the reference stimulus to yield robust effects.

At the neural level, EEG results show a clear signature of stimulus history, reflecting the encoding of past information into the responses to a current stimulus. Strikingly, our results show a neural signature that is more generalized compared with the behavioral effect. Brain responses to the reference stimulus indeed incorporate not only the “task-relevant” information yielding attractive effects (numerosity in the numerosity task, size in the size task), but a more complete representation of the different inducer dimensions. This suggests a partial dissociation between the neural encoding of stimulus history and the behavioral effect. Alternatively, the generalized neural effect might reflect influences that are actually occurring at the behavioral level but are too small to measure reliably. However, similarly to previous studies (Tsouli et al., 2019; Togoli et al., 2021), our paradigm was able to capture an opposite (repulsive) effect of duration on numerosity, with a similar effect size compared with the effect of numerosity. This suggests that a lack of sensitivity of the current paradigm to cross-dimensional biases is unlikely to explain the absence of those effects.

How can we explain such dissociation between behavioral and neural effects? First, our decoding procedure might capture a lingering trace of the past stimulus rather than the stimulus history information that affects perception/behavior. Although classification accuracies in some cases increase even before the stimulus onset, the temporal generalization pattern observed is not consistent with a lingering trace of the past stimulus. Second, another possibility concerns the effect of central tendency (e.g., Jazayeri and Shadlen, 2010), which is similarly based on stimulus history and has been recently linked to serial dependence (Tong and Dubé, 2022). However, since our analyses compared stimuli embedded in very similar temporal contexts, we believe that it is unlikely that central tendency contributed to the observed results. Finally, another possibility is that serial dependence would reflect only a subset of the past stimulus dimensions encoded in brain signals because of the involvement of an active gating mechanism. Namely, while the encoding of stimulus history carries a more complete representation of the past stimulus (similarly to the encoding of task-relevant and task-irrelevant stimulus features in working memory) (Bocincova and Johnson, 2019), the mechanisms involved in generating serial dependence effects would select and implement only the relevant information according to which dimension is highlighted by attention and/or task demands.

Although weaker, a consistent signature of stimulus history is also evident in the passive viewing paradigm of Experiment 2. This suggests that performing a specific task is not strictly necessary to observe such an effect (in line with previous results; Fornaciai and Park, 2018a), but that attention and/or task-related processes may play a role in modulating it. For instance, attention might explain a stronger effect at earlier latencies (i.e., via top-down modulation on early visual activity) (Somers et al., 1999; Grothe et al., 2018). On the other hand, the later amplification of CA observed in Experiment 1 is completely missing in the results of Experiment 2, suggesting that it is specifically related to performing an active task. A possibility is that this later peak might reflect the memory storage of information after decision-making, which could make the biased stimulus representation to be encoded in a more stable form compared with earlier perceptual processing (Oh et al., 2019).

Importantly, we observed a link between the serial dependence effect and the brain signals encoding past stimulus information. Indeed, at least in the numerosity and size task, the strength of the effect could be significantly predicted by the CA obtained in the decoding analysis. The timing of this significant relationship is early after the onset of the reference stimulus, starting at ∼35-65 ms and persisting until ∼250 ms. This in turn suggests that serial dependence emerges from early perceptual computations, potentially starting at the earliest levels of visual processing. Such an early correlate is consistent with previous studies proposing that the effect operates at the perceptual rather than decision-making level (Cicchini et al., 2017; Manassi et al., 2018; Collins, 2019; Manassi and Whitney, 2022). Our results provide direct evidence that early visual-evoked activity is indeed effectively linked to the behavioral effect.

If a link between the neural signature of stimulus history and the behavioral serial dependence effect emerges so early in the visual processing stream, why does serial dependence often show the properties of a high-level effect? Previous results indeed show that serial dependence is partially independent from the low-level features of the stimuli (Fischer et al., 2020), and works even across completely different stimuli (Fornaciai and Park, 2019b). Additionally, it often relies on prior choices rather than prior stimuli (e.g., Pascucci et al., 2019), and works according to the perceived rather than physical properties of a stimulus (Fornaciai and Park, 2021). An interesting possibility explaining this aspect of the effect is a dissociation between where the bias originates from and where it operates. Indeed, the bias itself may originate from high-level computations well before the onset of the current stimulus, and be transmitted back to earlier sensory stages via re-entrant feedback signals (Fornaciai and Park, 2019a, 2021), affecting perception directly. Our findings thus support the idea that, while serial dependence likely originates from high-level computations, it operates at an early processing stage biasing the phenomenological appearance of a stimulus rather than just how we judge or remember it (see also Collins, 2019; Manassi and Whitney, 2022).

Finally, it is interesting to note that serial dependence and its neural signature were measured in our paradigms on a very short timescale, with subsecond intervals across successive stimuli. Previous studies show serial dependence effects on a longer timescale spanning multiple seconds (e.g., Fischer and Whitney, 2014). Thus, an interesting question is whether a signature of serial dependence at early perceptual stages, like the one here, would be present for serial effects measured at different timescales. In general, we believe that the neural signature shown here should not depend on the timescale of the effect, but this remains an open question that requires dedicated experiments.

In conclusion, our findings highlight a set of important properties of serial dependence in magnitude perception and its neural signature. First, our results suggest the existence of an active mechanism gating past stimulus information according to its relevance. Second, we show that, while an active task is not necessary for the encoding of stimulus history in brain signals, performing a task amplifies this neural signature. Finally, we show a link between behavioral serial dependence effects and brain activity at very early latencies, suggesting that serial dependence emerges during early perceptual processing.
